# Optimization and Experimentation of Dual-Mass MEMS Gyroscope Quadrature Error Correction Methods

**DOI:** 10.3390/s16010071

**Published:** 2016-01-07

**Authors:** Huiliang Cao, Hongsheng Li, Zhiwei Kou, Yunbo Shi, Jun Tang, Zongmin Ma, Chong Shen, Jun Liu

**Affiliations:** 1 Key Laboratory of Instrumentation Science & Dynamic Measurement, Ministry of Education, North University of China, Tai Yuan 030051, China; caohuiliang@nuc.edu.cn (H.C.); kouzhiwei@imut.edu.cn (Z.K.); shiyunbo@nuc.edu.cn (Y.S.); tangjun16@126.com (J.T.); mzmncit@163.com (Z.M.); 2 Science and Technology on Electronic Test & Measurement Laboratory, North University of China, Tai Yuan 030051, China; 3 School of Instrument Science and Engineering, Southeast University, Nanjing 210096, China; hsli@seu.edu.cn

**Keywords:** dual-mass MEMS gyroscope, quadrature error correction, system stability, model simulation

## Abstract

This paper focuses on an optimal quadrature error correction method for the dual-mass MEMS gyroscope, in order to reduce the long term bias drift. It is known that the coupling stiffness and demodulation error are important elements causing bias drift. The coupling stiffness in dual-mass structures is analyzed. The experiment proves that the left and right masses’ quadrature errors are different, and the quadrature correction system should be arranged independently. The process leading to quadrature error is proposed, and the Charge Injecting Correction (CIC), Quadrature Force Correction (QFC) and Coupling Stiffness Correction (CSC) methods are introduced. The correction objects of these three methods are the quadrature error signal, force and the coupling stiffness, respectively. The three methods are investigated through control theory analysis, model simulation and circuit experiments, and the results support the theoretical analysis. The bias stability results based on CIC, QFC and CSC are 48 °/h, 9.9 °/h and 3.7 °/h, respectively, and this value is 38 °/h before quadrature error correction. The CSC method is proved to be the better method for quadrature correction, and it improves the Angle Random Walking (ARW) value, increasing it from 0.66 °/√h to 0.21 °/√h. The CSC system general test results show that it works well across the full temperature range, and the bias stabilities of the six groups’ output data are 3.8 °/h, 3.6 °/h, 3.4 °/h, 3.1 °/h, 3.0 °/h and 4.2 °/h, respectively, which proves the system has excellent repeatability.

## 1. Introduction

The precision of MEMS gyroscopes has improved a lot in the last decade, reaching a tactical grade level [1,2,3,4]. Being of small size, low cost and light weight, MEMS gyros have been applied to more and more areas, such as in inertial navigation, roller detection, automotive safety, industrial control, railway siding detection, consumer electronics and stability control systems [4,5,6]. During use, the acceleration along the sense axis causes great errors in the MEMS gyroscope output signal, and the dual-mass gyroscope structure effectively prevents this phenomenon from occurring by employing differential detection technology. Therefore, numerous research institutes are interested in this structure [1,7,8,9]. Meanwhile, low drift and low output error are required in many application areas, especially in μPNT systems [10,11], so, the dual-mass gyro bias drift suppression method is investigated in this work.

Most of the literature suggests that the dominant signal in output signals is the quadrature error, which is generated during the structure manufacturing process, and can produce an equivalent angular input of several hundred degrees per second [12,13,14,15,16]. The original source of quadrature is the coupling stiffness, which is modulated by drive mode movement and generates the quadrature error force. This force has the same frequency but a 90° phase difference with the Coriolis force, and stimulates the sense mode [14]. Most previous works utilize a phase-sensitivity demodulation method to extract the Coriolis signal from the sense channel [12,16], which requires accurate phase information and long term, full temperature range stability. However, demodulation phase errors and noise are usually present (sometimes more than 1° [12,15]) which creates undesirable bias. The coupling stiffness drift (the drive and sense modes’ equivalent stiffness vary with temperature and generate the coupling stiffness drift [7,14]) causes the quadrature error force drift, which is considered to be one of the most important reasons for bias long term drift, and has been proven [14,17] experimentally.

Previous works provide several effective ways to reduce quadrature error, and basically three approaches to reduce errors after the structure is manufactured [12,14] have been found: quadrature signal compensation, quadrature force correction and coupling stiffness correction. In [12], the quadrature error is reduced by DC voltage based on a synchronous demodulation and electrostatic quadrature compensation method, and the sigma-delta technology is employed in ADC and DAC. The research in [17] also employs a coupling stiffness correction method to improve the performance of a “butterfly” MEMS gyroscope. The bias stability and scale factor temperature stability is enhanced, increasing from 89 °/h and 662 ppm/°C to 17 °/h and 231 ppm/°C, respectively, which achieves the correction goal. The quadrature error correction in dual-mass tuning fork MEMS gyro structures is investigated in [14], and this work also proves that the quadrature stiffness is different in the left and right masses. A quadrature error correction closed loop is proposed in the work, which utilizes the coupling stiffness correction method. The masses are corrected separately. The stiffness correction combs utilize the unequal gap method with DC voltages [18]. The bias stability improves from 2.06 °/h to 0.64 °/h with the Allan Deviation analysis method, and the noise characteristics are also optimized [14]. Another coupling stiffness correction approach is proposed in [8]. In this work, a coupling stiffness correction controller uses PI technology, and the quadrature error equivalent input angular rate is measured as 450 °/s, and the experiment in the work shows that the bias stability and ARW improve from 7.1 °/h and 0.36 °/√h to 0.91 °/h and 0.034 °/√h, respectively. In [19], quadrature signal is compensated based on charge injecting technology in the sense loop, and the compensation signal has the same frequency, amplitude and anti-phase as the quadrature error signal. The quadrature error correction method proposed in [9] employs both the quadrature force and stiffness correction methods. The modulation reference signal is generated by PLL technology, and the correction loop uses a PI regulator. Here, the two masses are controlled together. A novel quadrature compensation method is proposed in [20] based on sigma-delta-modulators (ΣΔM), whereby the quadrature error is detected by utilizing a pure digital pattern recognition algorithm and is compensated by using DC bias voltages. Accordingly, the system works beyond the full-scale limits of the analog ΣΔM hardware. The quadrature error is compensated by an open loop charge injecting circuit in [21], whereby the circuit is implemented on ASIC and the experimental results show that the quadrature error component is effectively rejected. 

The work in this paper focuses on an optimization method for the dual-mass MEMS gyroscope quadrature error correction, and an investigation into the best way to improve the long term drift performance. The coupled MEMS gyroscope structure is introduced, and the quadrature error correction methods are investigated using two main approaches: the realization methods, including correction of two masses and separate correction; and the correction methods, including charge injecting correction, quadrature force correction and quadrature stiffness correction. Each correction method is demonstrated by theory analysis, model simulation and experiments, and, finally, the best optimization method is confirmed. This paper continues as follows: Section 2 introduces the dual-mass structure and the model including quadrature error. Section 3 analyzes three different correction methods and their simulations. Section 4 outlines the experiments and tests the above correction methods. Then, in Section 5, the general experiments of optimization quadrature error correction method are conducted and the reliability and repeatability of the system are analyzed. The results are discussed and concluding remarks are given in Section 6.

## 2. Dual Mass MEMS Gyroscope Structure and Quadrature Error Model 

### 2.1. Dual-Mass MEMS Gyroscope Structure

**Figure 1:**
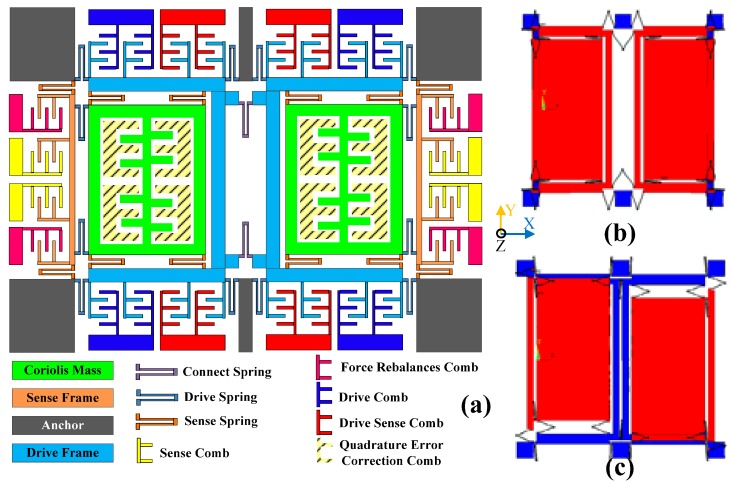
(**a**) Schematic and part photos of dual-mass gyroscope structure; (**b**) Drive anti-phase mode simulation; (**c**) Sense anti-phase mode simulation.

The dual-mass MEMS gyroscope researched in this paper is shown in Figure 1a. The structure is symmetrical, with a connecting spring. The drive mode is formed by a drive spring, drive frame, drive comb, connecting spring and drive sense comb. The sense mode is formed by a sense spring, sense frame, sense comb and sense feedback comb. The drive and sense springs support the entire active structure and decouple the drive and sense modes. The anchors are fixed with a glass bottom and support the springs. One side of the comb is connected with frames and moves with them, while the comb on the other side is fixed to a glass bottom and connected with metal lines to stimulate the capacitance or send the capacitance value signal to the interface. The left and right Coriolis masses generate the Coriolis force, and the quadrature error correction comb provides static electricity negative stiffness to compensate the quadrature coupling stiffness. The quadrature error correction combs are with unequal gaps and have been outlined in detail in the literature [14]. The drive anti-phase mode is shown in Figure 1b, and the sense anti-phase mode is shown in Figure 1c. The drive mode moves along the *X* axis while the sense mode vibrates along the *Y* axis. Since the left and right masses are coupled, the real working sense mode is the superposition of in-phase and anti-phase sense modes, but the anti-phase mode dominates the sense mode movement, and is the focus of the discussion in this work.

### 2.2. Quadrature Error Model

The movement equation with coupling stiffness and coupling damping of the gyroscope structure can be expressed as [14]:

(1) $$\left[\begin{array}{l} m_{x}\ddot{x}\\ m_{y}\ddot{y} \end{array}\right]+\left[\begin{array}{cc} c_{xx} & c_{xy}\\ c_{yx} & c_{yy} \end{array}\right]\left[\begin{array}{l} \dot{x}\\ \dot{y} \end{array}\right]+\left[\begin{array}{cc} k_{xx} & k_{xy}\\ k_{yx} & k_{yy} \end{array}\right]\left[\begin{array}{l} x\\ y \end{array}\right]=\left[\begin{array}{l} \;F_{dx}\\ -2m_{c}\Omega_{z}\dot{x} \end{array}\right]$$

where *m_x_* and *m_y_* are equivalent masses of drive and sense modes, respectively; *c_xx_*, *c_yy_* and *k_xx_*, *k_yy_* are effective damping and stiffness of drive and sense modes; *c_xy_* and *c_yx_* are coupling damping between drive and sense modes; *k_xy_* and *k_yx_* are coupling stiffness caused by machining error; *x* and *y* are the displacements of drive and sense frames; *F_dx_* is drive force; *m_c_* is Coriolis mass and *m_c_* ≈ *m_y_*; *Ω_z_* is the angular rate input around *z* axis. The stiffness elements in Equation (1) can be calculated by:

(2) $$\left\{ \begin{array}{l} k_{xx}=k_{x}\cos^{2}\beta_{Qx}+k_{y}\sin^{2}\beta_{Qy}\\ k_{xy}=k_{yx}=k_{x}\sin\beta_{Qx}\cos\beta_{Qx}-k_{y}\cos\beta_{Qy}\sin\beta_{Qy}\\ k_{yy}=k_{x}\sin^{2}\beta_{Qx}+k_{y}\cos^{2}\beta_{Qy} \end{array}\right.$$

where *k_x_* and *k_y_* are the design stiffness along designed axes *x* and *y*; *β_Qx_* and *β_Qy_* are the quadrature error angle and they are the angles between practical axes and designed axes [14,22], and usually it is assumed that *β_Qx_* = *β_Qy_*. So, sense mode movement can be expressed with the following equation:

(3) $$\begin{array}{l} m_{y}\ddot{y}+c_{yy}\dot{y}+\left(k_{x}\sin^{2}\beta_{Qx}+k_{y}\cos^{2}\beta_{Qx}\right)y\\ =\underset{\begin{array}{l} Coupling\;stiffness\;force\;F_{QE}\\ \;(Quadrature\;error) \end{array}}{\underset{︸}{\frac{1}{2}\left(k_{y}-k_{x}\right)\sin2\beta_{Qx}x}}-\underset{\begin{array}{l} Coriolis\;force\\ \;F_{C} \end{array}}{\underset{︸}{2m_{c}\Omega_{z}\dot{x}}}-\underset{\begin{array}{l} Coupling\;damping\;\\ \;force\;F_{CD}\; \end{array}}{\underset{︸}{c_{yx}\dot{x}}} \end{array}$$

The force can be divided into three parts: coupling stiffness force (quadrature error force) *F_QE_*, Coriolis force *F_C_* and coupling damping force *F_CD_*. Since the drive mode movement can be described as *x* = *A_x_*cos(*ω_d_*t), the *F_C_* and *F_CD_* are with same phase, and *F_QE_* has a 90 degree phase difference with them. Although *F_CD_* cannot be eliminated, high vacuum packaging is an effective method to decrease *c_yx_* and *F_CD_*. The mechanical parameters of the MEMS gyroscope are listed in Table 1. Substituting the parameter values into Equation (2), we can calculate that the quadrature error angle *β_Qx_* is 0.15° approximately. Furthermore, the amplitude of *F_QE_* (2.68 × 10^−7^ N and the equivalent input angular rate *Ω_QE_* is 200 °/s) is over 60 times larger than *F_CD_* (2.68 × 10^−9^ N, the equivalent input angular rate *Ω_CD_* is 3.3 °/s), and *F_QE_* governs the sense mode force.

**Table 1:** MEMS gyroscope parameter values.

Parameter	Value
*ω_d_*	3488.9 × 2π rad/s
*k_x_*	593.9 N/m
*k_y_*	532.0 N/m
*k_yx_, k_xy_*	0.1719 N/m
*c_yx_*	1.29 × 10^−7^ N/m/s
*A_x_*	1.56 μm

The sense loop employs phase sensitivity detection technology to pick up the Coriolis signal, and, in real working conditions, the demodulation reference signal is *V_dem_*sin(*ω_d_*t + *φ_e_* + *φ_n_*), and *φ_e_*, *φ_n_* are phase error and phase noise, respectively. The parameters *Ω_QE_*, *Ω_CD_* and *Ω_IP_* are the equivalent input angular rates of quadrature error, coupling damping error and the real input angular rate, respectively, and neglecting the other errors and noise, the gyro output angular rate value *Ω_OP_* can be expressed as [15]:

(4) $$\Omega_{OP}\approx \left(\Omega_{IP}+\Omega_{CD}\right)\cos(\varphi_{e}+\varphi_{n})+\Omega_{QE}\sin(\varphi_{e}+\varphi_{n})$$

**Figure 2:**
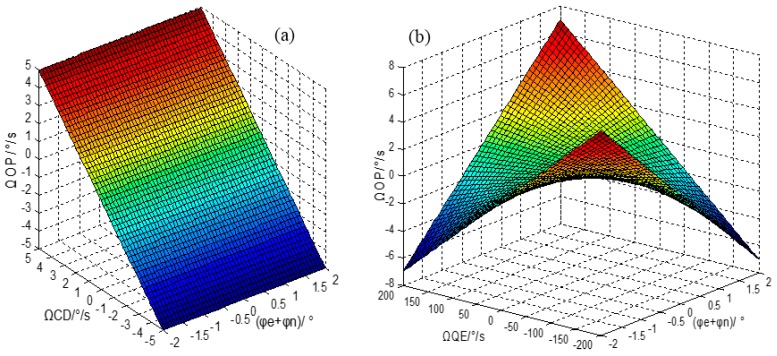
(**a**) The relationship between *Ω_OP_*, *φ_e_* + *φ_n_* and *Ω_CD_*; (**b**) The relationship between *Ω_OP_*, *φ_e_* + *φ_n_* and *Ω_QE_.*

We assume |*Ω_QE_*| < 200 °/s, |*Ω_CD_*| < 5 °/s, |*φ_e_* + *φ_n_*| < 2°, and let *Ω_IP_* = 0°/s. Figure 2a shows the curve of the relationship of *Ω_OP_*, *Ω_CD_* and *φ_e_* + *φ_n_* and Figure 2b shows the curve of the relationship of *Ω_OP_*, *Ω_QE_* and *φ_e_* + *φ_n_*. It is obvious that the *Ω_QE_* generates 7 °/s *Ω_OP_* signal when *Ω_QE_* = 200 °/s, *Ω_CD_* is almost proportional to *Ω_OP_*, and the demodulation phase error does not have much influence, so, briefly speaking, the *Ω_QE_* provides bias to *Ω_OP_*, and *Ω_CD_* is the dominated element of *Ω_OP_* when *Ω_CD_* is large enough. What is worse is that *Ω_QE_* changes with temperature and structure vibration stiffness, so the simple open loop compensation for *Ω_QE_* is not satisfactory. Therefore, the optimization quadrature error correction system for this gyro structure should be investigated.

### 2.3. Dual-Mass Quadrature Error Model

**Figure 3:**
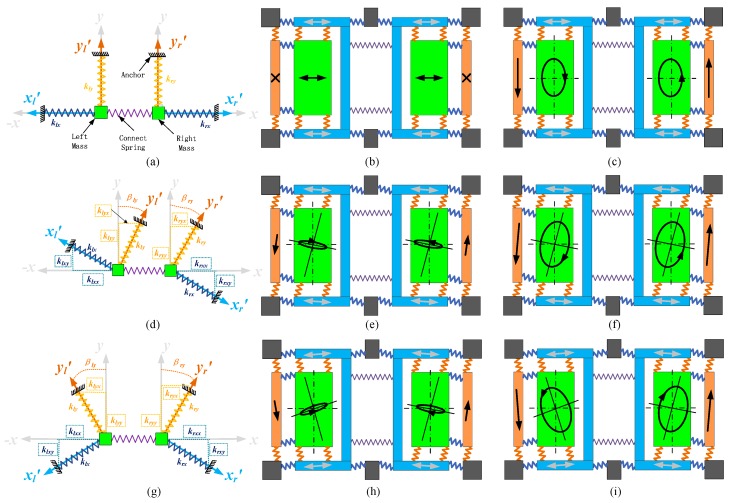
(**a**) Stiffness system of ideal dual-mass gyro structure; (**b**) Ideal structure movement without angular rate input; (**c**) Ideal structure movement with constant angular rate input; (**d**) Stiffness system with in-phase quadrature error angular structure; (**e**) The movement of in-phase quadrature error angular structure without angular rate input; (**f**) The movement of in-phase quadrature error angular structure with constant angular rate input; (**g**) Stiffness system with anti-phase quadrature error angular structure; (**h**) The movement of anti-phase quadrature error angular structure without angular rate input; (**i**) The movement of anti-phase quadrature error angular structure with constant angular rate input.

Figure 3 shows the equivalent stiffness and masses system and structure motion of the dual-mass gyro structure. The design drive and sense stiffness axis are *x* and *y* (gray); the real drive and sense axis of left and right masses after manufacturing are *x_l_’*, *x_r_’* (light blue color) and *y_l_’*, *y_r_’* (light yellow color), respectively; the stiffness of drive and sense modes of left and right masses after manufacture are *k_lx_*, *k_rx_* (dark blue color) and *k_ly_*, *k_ry_* (dark yellow color), respectively; the projections of *k_lx_* on *−x* and *y* axis are *k_lxx_* and *k_lxy_*; the projections of *k_rx_* on *x* and *y* axis are *k_rxx_* and *k_rxy_*; the projections of *k_ly_* on *−x* and *y* axis are *k_lyx_* and *k_lyy_*; the projections of *k_ry_* on *x* and *y* axis are *k_ryx_* and *k_ryy_*; the quadrature error angular of left and right masses are *β_ly_* and *β_ry_*. 

**Figure 4:**
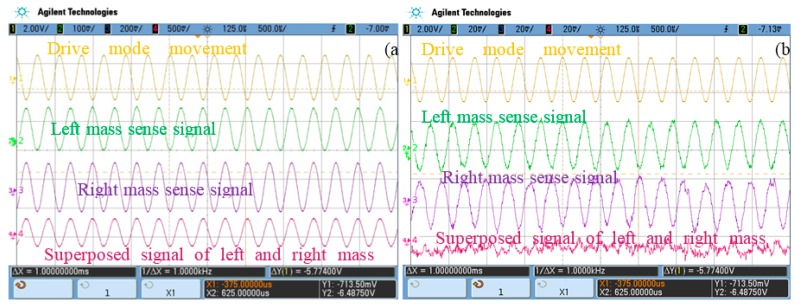
(**a**) Test signals before quadrature error correction; (**b**) Test signals after quadrature error using the correction method with the two masses together.

In the design stage, |*k_lx_*| = |*k_rx_*|, |*k_ly_*| = |*k_ry_*|, and *β_ly_* = *β_ry_* = 0, but after the manufacturing process, the parameters change and they do not fit with the equations, so the coupling stiffness of the two masses is different. The drive-mode movement signal (channel 1), left and right masses sense signals (channels 2 and 3) and their superposed signal (channel 4) before quadrature error correction are tested and shown in Figure 4a. The left and right mass sense signals are of the same phase and frequency, and they are anti-phase with drive mode movement signals, which means that the main element in sense signals is quadrature error. The left and right mass sense signal amplitudes are 150 mV and 300 mV, respectively, which proves that the two masses’ quadrature errors are different. 

### 2.4. Dual-Mass Gyro Quadrature Error Correction Realization Methods

Two realization methods for dual-mass gyro quadrature error correction are discussed in this work: two masses corrected together and, two masses corrected separately. The “correction together” method entails providing the same correction signals to both masses and observing the two masses’ sense superposed signals. This method is tested with the CSC method, and the corresponding oscilloscope signals are shown in Figure 4b. It is obvious that the superposed signal does not have any quadrature error characteristics but noises. However, the left and right mass sensing signals are in-phase and anti-phase with the drive-mode movement signal, which indicates that two masses’ sense signals are still governed by quadrature error, and their amplitudes are nearly the same at about 35 mV. Although the “correction together” method removes more than 80% of the quadrature error, this method cannot eliminate quadrature error totally, and one mass (the left mass in Figure 4) is overcorrected. Therefore, each mass should be corrected separately to make sure its quadrature error is completely corrected.

## 3. Dual-Mass Gyro Quadrature Error Correction Method 

### 3.1. Quadrature Error Correction Methods and Gyro System

**Figure 5:**
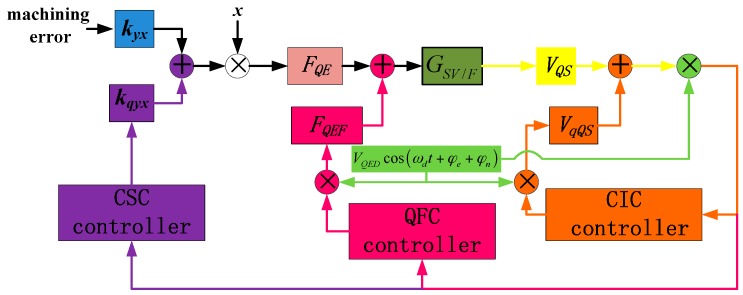
Diagram of quadrature error generating process and correction method.

From the above content, it can be concluded that the quadrature error is generated by the coupling stiffness *k_yx_*, which is modulated by drive mode movement and produces the quadrature error force *F_QE_*. The force stimulates the sense mode and quadrature error displacement occurs. This leads directly to a quadrature error signal in the sense loop, and after the occurrence of phase error in the demodulator, the quadrature error signal becomes one part of the gyro output. Consequently, this work divides this process into three stages as Figure 5 shows: the quadrature error stiffness stage, the quadrature error force stage and the quadrature error signal stage.

Three different correction methods are proposed to deal with the three stages individually [14]:

- The charge injecting correction (CIC) method focuses on the quadrature error signal *V_QS_* in the sense loop, and does not influence the movement of the gyro structure.
- The quadrature force correction (QFC) method takes quadrature error force *F_QE_* as the controlling object, and does not change the stiffness of the structure.
- The coupling stiffness correction (CSC) method aims at the quadrature error coupling stiffness directly, and in theory, this method eliminates quadrature error fundamentally.

**Figure 6:**
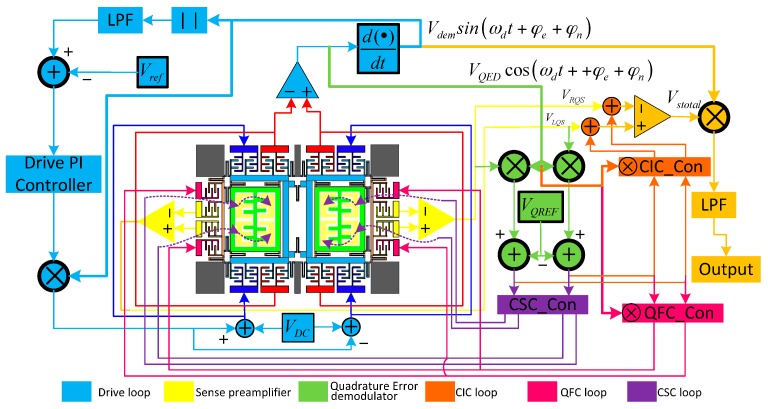
Diagram of the gyro monitoring system.

The monitoring system of the gyro is shown in Figure 6. The drive loop employs a self-oscillation method using an AGC controller [23]. The drive-mode movement signals are picked up from left and right drive sense combs, and the displacement signals are transformed into electric signals with a differential instrument pre-amplifier. Its output signal has the same phase as the drive movement signal and is utilized for quadrature error demodulation. The phase of this signal is shifted 90 degrees and generates *V_dem_*sin(*ω_d_*t + *φ_e_* + *φ_n_*), which is used for Coriolis in-phase signal demodulation. The left and right sense frame displacements are picked up individually with a differential instrument pre-amplifier, and the output signals of sense pre-amplifiers are demodulated separately to achieve their own quadrature error values. The working principle and subquadrature error correction systems will be expanded in this section.

### 3.2. Charge Injecting Correction Method and System

**Figure 7:**
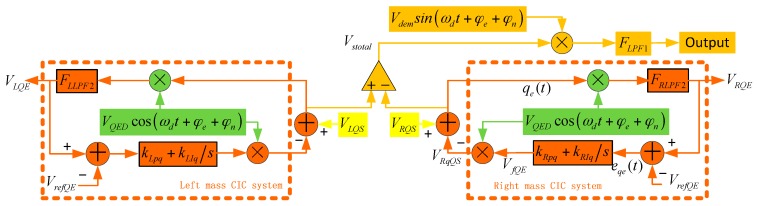
Dual-mass structure CIC system.

The controlling object of CIC is the quadrature error electrical signal in the sense loop, and this method does not require structure cooperation, so it is fit for simple structures and sense open loops. In [16], this method is utilized to eliminate the quadrature signal and observe the Coriolis same-frequency signal, and good results were achieved. In Figure 5, the quadrature error signal *V_QS_* is demodulated by *V_QED_*cos(*ω_d_*t + *φ_e_* + *φ_n_*), *V_QED_* = 1.5 V, and the quadrature error amplitude (QEA) is picked up (because of *φ_e_* + *φ_n_*, QEA contains demodulation error). The two masses’ QEA are expressed as *V_LQE_* and *V_RQE_*. The CIC system diagram is shown in Figure 7. The left and right mass CIC systems share the same demodulation signal and comparator signal *V_refQE_ = 0*. The right mass CIC system is analyzed as a sample to investigate the system stability. The cut-off frequency, quality factor and gain of *F_RLPF2_* are 200 Hz, 1 and –10.

The right mass sense pre-amplifier output signal *V_RQS_* contains the Coriolis signal *V_sC_*, stiffness coupling signal *V_sMk_*, damping coupling signal *V_sMc_*, electrical cross-coupling signal *V_sE_*, force cross-coupling signal *V_sF_* and other elements [12], and can be expressed as:

(5) $$\left[\begin{array}{l} V_{sC}\\ V_{sMk}\\ V_{sMc}\\ V_{sE}\\ V_{sF} \end{array}\right]=\left[\begin{array}{l} 2\Omega_{z}A_{x}\omega_{d}m_{c}G_{sV/F}\sin(\omega_{d}t)\\ \alpha_{sMk}A_{x}G_{sV/F}\cos(\omega_{d}t)\\ \alpha_{sMc}A_{x}\omega_{d}G_{sV/F}\sin(\omega_{d}t)\\ \alpha_{sE}V_{ac}\sin(\omega_{d}t+\theta_{sE})\\ \alpha_{sF}F_{d}G_{sV/F}\sin(\omega_{d}t+\theta_{sF}) \end{array}\right]$$

where *G_sV/F_* is the force-voltage transform function of gyro structure; *α_sMk_* and *α_sMc_* are constant and are involved with coupling stiffness and damping; *α_sE_* and *θ_sE_* are the amplitude and phase difference of electrical cross-coupling signal; *V_ac_* is drive mode stimulating AC voltage amplitude; *α_s__F_* and *θ_s__F_* are the amplitude and phase difference of force cross-coupling signal. Then, ignoring the influence of *θ_sE_* and *θ_s__F_*, and *V_RQS_* can be written as:

(6) $$\begin{array}{l} V_{RQS}=V_{sC}+V_{sMk}+V_{sMc}+V_{sE}+V_{sF}\\ =\left[(2\Omega_{z}m_{c}+\alpha_{sMc})A_{x}\omega_{d}G_{sV/F}+\alpha_{sE}V_{ac}+\alpha_{sF}F_{d}G_{sV/F}\right]\sin(\omega_{d}t)+\alpha_{sMk}A_{x}G_{sV/F}\cos(\omega_{d}t) \end{array}$$

In Figure 7, we have:

(7) $$\begin{array}{l} V_{RQE}=\left[q_{e}V_{QED}\cos(\omega_{d}t+\varphi_{e}+\varphi_{n})\right]{|}_{F_{LPF2}}\\ \;=\left[\left(V_{RQS}-V_{fQE}V_{QED}\cos(\omega_{d}t+\varphi_{e}+\varphi_{n})\right)V_{QED}\cos(\omega_{d}t+\varphi_{e}+\varphi_{n})\right]{|}_{F_{LPF2}} \end{array}$$

(8) $$e_{qe}(t)=V_{RQE}(t)-V_{refQE}(t)$$

(9) $$V_{fQE}(t)=e_{qe}(t)k_{Rpq}+k_{RIq}\int_{0}^{t}e_{qe}(t)dt$$

Combining Equations (6) and (7), and filtering the high frequency element, we can rewrite Equation (7) as:

(10) $$\begin{array}{l} V_{RQE}=\frac{V_{QED}}{2}\times \{\alpha_{sMk}A_{x}G_{sV/F}\cos(\varphi_{e}+\varphi_{n})-V_{fQE}V_{QED}-\\ \;\left[(2\Omega_{z}m_{c}+\alpha_{sMc})A_{x}\omega_{d}G_{sV/F}+\alpha_{sE}V_{ac}+\alpha_{sF}F_{d}G_{sV/F}\right]\sin(\varphi_{e}+\varphi_{n})\}\\ \;\approx \frac{V_{QED}\left(\alpha_{sMk}A_{x}G_{sV/F}-V_{fQE}V_{QED}\right)}{2}=\frac{A_{QE}V_{QED}-V_{fQE}{V_{QED}}^{2}}{2} \end{array}$$

where *A_QE_* is the quadrature error in-phase element in *V_RQE_* signal. Combining Equations (8) and (9), it can be found that:

(11) $$\frac{V_{RQE}}{A_{QE}}=\frac{sV_{QED}}{2s+\left(k_{Rpq}s+k_{RIq}\right){V_{QED}}^{2}}$$

There is one pole in Equation (11):

(12) $$p_{RCIC}=-\frac{2+k_{Rpq}{V_{QED}}^{2}}{{V_{QED}}^{2}k_{RIq}}$$

Also, *k_Rpq_ = 0.2*, *k_RIq_ = 2000*, so *p_RCIC_ < 0*, the system is stable. When *s = 0*, the system is in a static state, then:

(13) $$A_{QE}\approx {V_{RQE}\left(k_{Rpq}+\frac{k_{RIq}}{s}\right)V_{QED}|}_{s=0}=V_{fQE}V_{QED}$$

Then, the quadrature error (cosine) component in *q_e_(t)* is:

(14) $$q_{e\cos}(t)\approx A_{QE}\cos(\omega_{d}t)-V_{fQE}V_{QED}\cos\left(\omega_{d}t+\varphi_{e}+\varphi_{n}\right)\approx 0$$

**Figure 8:**
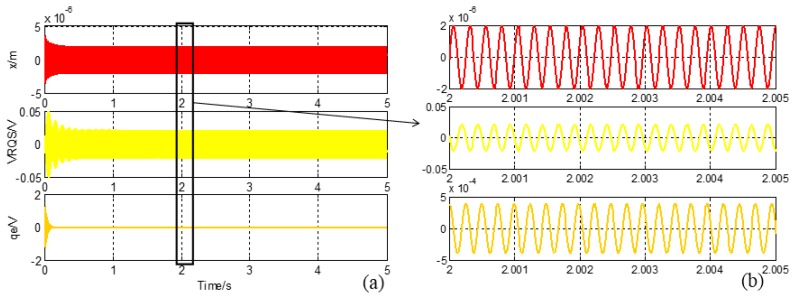
(**a**) Right-mass CIC system simulation curves; (**b**) Enlarged curves of right-mass CIC system simulation result.

It is obvious from Equation (14) that the quadrature error component is basically eliminated. The system is simulated in Simulink soft, and the curves are shown in Figure 8: the first curve is the drive-mode motion *x*, the second and the third curves are the right-mass sense signal *V_RQS_* and after quadrature error correction signal *q_e_*. The gyro is started at 0 s, and the drive-mode is steady after 0.2 s, while the amplitude is about 1 μm. The CIC system is in a stable state after 0.5 s, the curves indicate that *V_RQS_* has a 180 degree phase-difference with drive-mode movement, so the quadrature error signal is the main component of *V_RQS_*. The peak-peak amplitude of *q_e_* value is 0.8 mV and its phase is the same as Coriolis signal, which proves that the quadrature error signal is compensated completely.

### 3.3. Quadrature Force Correction Method and System

**Figure 9:**
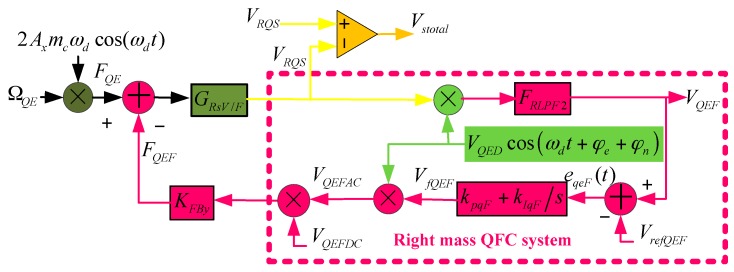
Right mass QFC system.

The QFC method requires the sense feedback comb to generate electrostatic force and balance the quadrature error force. Its controlling object is *F_QE_* in Figure 5. This method is usually utilized with a sense closed loop, and it restrains the sense mode quadrature error motion, so it is better than the CIC method in theory. However, QFC method needs the modulation step, which is also influenced by phase error elements. Similarly to the CIC method, the whole QFC system also has two sub systems: left and right masses systems. This paper takes the right mass QFC system as an example to illustrate QFC working principles and system stabilization. The right mass QFC system is shown in Figure 9. Since QFC changes force, this paper divides *G_sV/F_* into two parts, and this section discusses the right mass part *G_RsV/F_*. Also, the low pass filter is the same one as in the CIC system. In order to make the analysis process clear, the quadrature error force is expressed by *Ω_QE_*. The voltage and force transform coefficient is *K_FBy_* = 1.487 × 10^-7^ N/V. The electrostatic force is generated by DC (*V_QEFDC_* = 5 V) and AC (*V_QEFAC_*) power, and the force is proportional to their product. The DC power is constant and AC power is controlled to achieve accurate correction force. The refrernce voltage *V_refQEF_* = 0V, and *k_pqF_* = 0.01, *k_IqF_* = 1000.

From Figure 9, the equations below can be obtained:

(15) $$F_{QE}=2\Omega_{QE}A_{x}m_{c}\omega_{d}\cos(\omega_{d}t)$$

(16) $$F_{QEF}=K_{FBy}V_{fQEF}V_{QEFDC}V_{QED}\cos(\omega_{d}t+\varphi_{e}+\varphi_{n})$$

(17) $$V_{RQS}=\left(F_{QE}-F_{QEF}\right)G_{RsV/F}$$

(18) $$V_{QEF}=V_{RQS}V_{QED}\cos(\omega_{d}t+\varphi_{e}+\varphi_{n})F_{RLPF2}$$

Then, after Laplace Transform, Equations (15), (16) and (18) can be rewritten as:

(19) $$F_{QE}\left(s\right)=A_{x}m_{c}\omega_{d}\left[\Omega_{QE}\left(s+j\omega_{d}\right)+\Omega_{QE}\left(s-j\omega_{d}\right)\right]$$

(20) $$F_{QEF}\left(s\right)=\frac{1}{2}K_{FBy}V_{QEFDC}\left[V_{fQEF}\left(s+j\omega_{d}\right)e^{-j(\varphi_{e}+\varphi_{n})}+V_{fQEF}\left(s-j\omega_{d}\right)e^{j(\varphi_{e}+\varphi_{n})}\right]$$

(21) $$\begin{array}{l} V_{QEF}(s)=\{\left[F_{QE}\left(s+j\omega_{d}\right)-F_{QEF}\left(s+j\omega_{d}\right)\right]G_{RsV/F}\left(s+j\omega_{d}\right)e^{-j(\varphi_{e}+\varphi_{n})}\\ \;+\left[F_{QE}\left(s-j\omega_{d}\right)-F_{QEF}\left(s-j\omega_{d}\right)\right]G_{RsV/F}\left(s-j\omega_{d}\right)e^{j(\varphi_{e}+\varphi_{n})}\}V_{QED}F_{RLPF2}(s) \end{array}$$

Substituting Equations (19) and (20) into Equation (21), considering the low-pass filter, and ignoring the double frequency elements, we can rewrite Equation (21) as:

(22) $$\begin{array}{l} V_{QEF}(s)=\{\left[A_{x}m_{c}\omega_{d}\Omega_{QE}\left(s\right)e^{-j(\varphi_{e}+\varphi_{n})}-\frac{1}{2}K_{FBy}V_{QEFDC}V_{fQEF}V_{QED}\left(s\right)\right]G_{RsV/F}\left(s+j\omega_{d}\right)+\\ \;\left[A_{x}m_{c}\omega_{d}\Omega_{QE}\left(s\right)e^{j(\varphi_{e}+\varphi_{n})}-\frac{1}{2}K_{FBy}V_{QEFDC}V_{QED}V_{fQEF}\left(s\right)\right]G_{RsV/F}\left(s-j\omega_{d}\right)\}V_{QED}F_{RLPF2}(s)\\ =\{\left[A_{x}m_{c}\omega_{d}\Omega_{QE}\left(s\right)\cos((\varphi_{e}+\varphi_{n}))-\frac{1}{2}K_{FBy}V_{QEFDC}V_{QED}V_{fQEF}\left(s\right)\right]\left[G_{RsV/F}\left(s+j\omega_{d}\right)+G_{RsV/F}\left(s-j\omega_{d}\right)\right]+\\ \;jA_{x}m_{c}\omega_{d}\Omega_{QE}\left(s\right)\sin(\varphi_{e}+\varphi_{n})\left[G_{RsV/F}\left(s-j\omega_{d}\right)-G_{RsV/F}\left(s+j\omega_{d}\right)\right]\}V_{QED}F_{RLPF2}(s) \end{array}$$

It is shown from the above equation that *φ_e_* + *φ_n_* introduces undesirable items, and we consider it is a small value (usually less than 2 degrees) and gradually-varying parameter, which generates bias and long-term drift but does not influence the QFC system’s stability. So, we assume *φ_e_* + *φ_n_* = 0°, and simplify Equation (22) as:

(23) $$\begin{array}{l} V_{QEF}(s)\approx \left[A_{x}m_{c}\omega_{d}\Omega_{QE}\left(s\right)-\frac{1}{2}K_{FBy}V_{QEFDC}V_{QED}V_{fQEF}\left(s\right)\right]\times \\ \;\left[G_{RsV/F}\left(s+j\omega_{d}\right)+G_{RsV/F}\left(s-j\omega_{d}\right)\right]V_{QED}F_{RLPF2}(s) \end{array}$$

Also, in Figure 9, we have:

(24) $$V_{fQEF}(s)=\left(V_{QEF}(s)-V_{refQEF}\right)\left(k_{pqF}+\frac{k_{IqF}}{s}\right)$$

Combining Equations (23) and (24), we can find that:

(25) $$\frac{V_{fQEF}\left(s\right)}{\Omega_{QE}\left(s\right)}\approx \frac{2A_{x}m_{c}\omega_{d}V_{QED}F_{RLPF2}(s)\left[G_{sV/F}\left(s+j\omega_{d}\right)+G_{sV/F}\left(s-j\omega_{d}\right)\right]\left(k_{pqF}+\frac{k_{IqF}}{s}\right)}{1+K_{FBy}V_{QEFDC}{V_{QED}}^{2}F_{RLPF2}(s)\left[G_{sV/F}\left(s+j\omega_{d}\right)+G_{sV/F}\left(s-j\omega_{d}\right)\right]\left(k_{pqF}+\frac{k_{IqF}}{s}\right)}$$

When the quadrature error equivalent angular rate is constant, s = 0, and the system is under stable conditions, then in Equation (25), we have:

(26) $$1<<{K_{FBy}V_{QEFDC}{V_{QED}}^{2}F_{RLPF2}(s)\left[G_{sV/F}\left(s+j\omega_{d}\right)+G_{sV/F}\left(s-j\omega_{d}\right)\right]\left(k_{pqF}+\frac{k_{IqF}}{s}\right)|}_{s=0}$$

**Figure 10:**
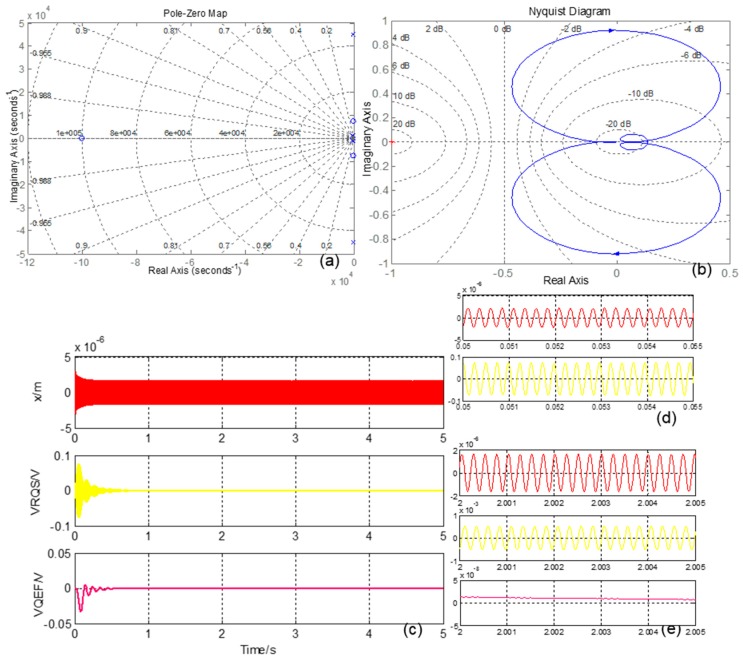
(**a**) QFC system Pole-Zero map; (**b**) QFC system Nyquist Diagram; (**c**) QFC system simulation curves; (**d**) QFC system start-up stage enlarged curves; (**e**) QEF system stable stage enlarged curves.

Then, Equation (25) can be expressed as:

(27) $$V_{fQEF}\approx \frac{2A_{x}m_{c}\omega_{d}}{K_{FBy}V_{QEFDC}V_{QED}}\Omega_{QE}$$

Equation (27) shows that the controlling value is proportional to the quadrature error equivalent angular rate, and the coefficient can be adjusted by drive-mode amplitude, force generation DC power and demodulation signal amplitude. The QFC system is analyzed in the Simulink software, and the results are shown in Figure 10. In the Pole-Zero Map, all poles are distributed in the negative real axis, and, in the Nyquist Diagram, the curve does not contain a (−1, 0j) point, which indicates that the system is stable. The curves in Figure 10c show that in the start-up stage (*t* < 0.1 s), *V_RQS_* is anti-phase with drive-mode movement, and this means the quadrature error signal is the dominating signal. After *t* = 0.7 s, the QFC system is in a stable stage, and *V_RQS_’s* phase has a 90 degree difference with drive-mode movement, which shows that the quadrature error force is basically corrected.

### 3.4. Coupling Stiffness Correction Method and System

**Figure 11:**
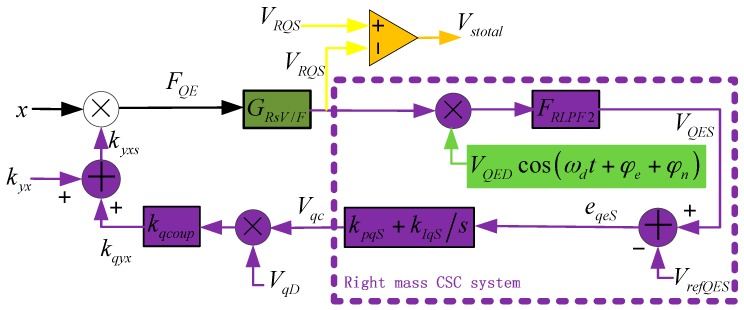
Right mass SCS system.

The CSC method utilizes quadrature error correction combs to generate negative electrostatic stiffness, and corrects quadrature error coupling stiffness. This special comb is an unequal gap style, and is introduced in [14,18]. Its stiffness is expressed as:

(28) $$k_{qxy}=k_{qyx}=k_{qcoup}V_{qD}V_{qc}=-\frac{4n_{q}\varepsilon_{0}h}{{y_{q0}}^{2}}\left(1-\frac{1}{\lambda^{2}}\right)V_{qD}V_{qc}$$

where, *k_qxy_* and *k_qyx_* are the quadrature error correction comb stiffness along designed axes *x* and *y*; *k_qcoup_* = −0.0049 N/m/V^2^ is the stiffness coefficient of correction comb; *V_qD_* = 5 V and *V_qc_* are the correction fixed voltage and controlling voltage; *n_q_* = 45 is the number of combs; *ε_0_* = 8.85 × 10^−12^ F/m is the permittivity of vacuum; *h =* 60 μm is the thickness of the comb; *y_q0_* = 4 μm and *x_0_* are the parallel capacitance’s gap and overlap length, respectively; *λ* = 2.4 is a constant. Figure 11 shows the right mass CSC system which is the same as the left mass CSC system. In this figure, the coupling stiffness *k_yx_* is modulated by drive-mode movement, and the controller employs PI technology. The correction stiffness does not need to be modulated by quadrature error in-phase signal, which is better for circuit simplification and power consumption.

Like the analysis process in the previous section, we can create the equations below:

(29) $$V_{RQS}=F_{QE}G_{RsV/F}=x\left(k_{yx}+k_{qyx}\right)G_{RsV/F}$$

(30) $$V_{qc}=\left(V_{QES}-V_{refQES}\right)\left(k_{pqS}+\frac{k_{IqS}}{s}\right)$$

We have *x = A_x_cos(ω_d_t)* and *V_refQES_ = 0*, after the low-pass filter, *V_QES_* can be expressed as:

(31) $$V_{QES}=\frac{1}{2}A_{x}\left(k_{yx}+k_{qyx}\right)G_{RsV/F}V_{QED}\cos\left(\varphi_{e}+\varphi_{n}\right)$$

Then, combining Equations (28), (30) and (31), we have:

(32) $$\begin{array}{l} \text{ ​​}\frac{k_{qyx}\left(s\right)}{k_{yx}\left(s\right)}=\\ \frac{\frac{A_{x}}{2}k_{qcoup}V_{qD}V_{QED}F_{RLPF2}(s)\left[G_{RsV/F}\left(s+j\omega_{d}\right)+G_{RsV/F}\left(s-j\omega_{d}\right)\right]\cos\left(\varphi_{e}+\varphi_{n}\right)\left(k_{pqS}+\frac{k_{IqS}}{s}\right)}{1-\frac{A_{x}}{2}k_{qcoup}V_{qD}V_{QED}F_{RLPF2}(s)\left[G_{RsV/F}\left(s+j\omega_{d}\right)+G_{RsV/F}\left(s-j\omega_{d}\right)\right]\cos\left(\varphi_{e}+\varphi_{n}\right)\left(k_{pqS}+\frac{k_{IqS}}{s}\right)} \end{array}$$

When the system is under stable state, s = 0, we design the PI controller parameters as *k_pqS_* = 0.01, *k_IqS_* = 2,500,000, and the above equation has:

(33) $$1<<{\frac{A_{x}}{2}k_{qcoup}V_{qD}V_{QED}F_{LPF2}(s)\left[G_{RsV/F}\left(s+j\omega_{d}\right)+G_{RsV/F}\left(s-j\omega_{d}\right)\right]\cos\left(\varphi_{e}+\varphi_{n}\right)\left(k_{pqS}+\frac{k_{IqS}}{s}\right)|}_{s=0}$$

Then, it can be found that:

(34) $$k_{qyx}\approx -k_{yx}$$

**Figure 12:**
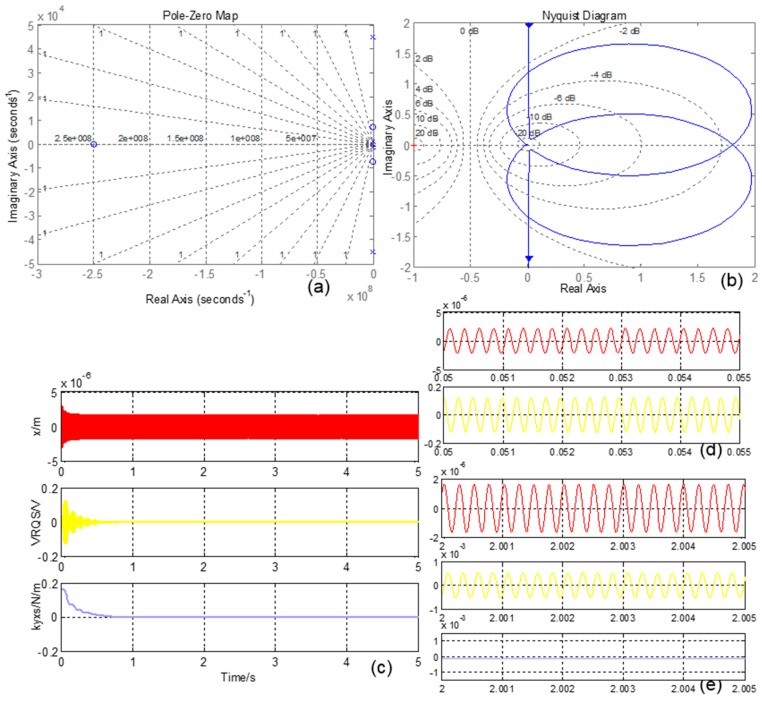
(**a**) CSC system Pole-Zero map; (**b**) CSC system Nyquist Diagram; (**c**) CSC system simulation curves; (**d**) CSC system start-up stage enlarged curves; (**e**) CSF system stable stage enlarged curves.

The coupling stiffness is corrected. The CSC system is simulated and the curves are shown in Figure 12. The Pole-Zero Map is shown in Figure 12a, and no pole is in the positive real axis, which means the system is stable. Figure 12b is the Nyquist Diagram, and the curve does not contain a (−1, 0j) point, which also proves the system’s stability. The time domain simulation curves are shown in Figure 12c,d,f, and the curves indicate that the CSC system is in a stable state after about 0.7 s. It is obvious that in the start-up stage, the VRQS signal is mainly consisted of the quadrature error signal. However, in a stable state, the dominate element is the Coriolis in-phase signal. Furthermore, the overall coupling stiffness *k_yxs_* is suppressed from its original value (about 0.18 N/m) to −0.00016 N/m, and *k_yx_* is basically corrected, which proves Equation (34).

## 4. Experimental Section and Discussion 

**Figure 13:**
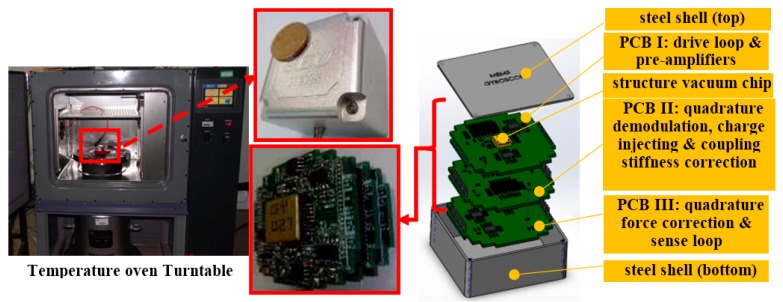
Experimental equipment and gyro section view.

**Figure 14:**
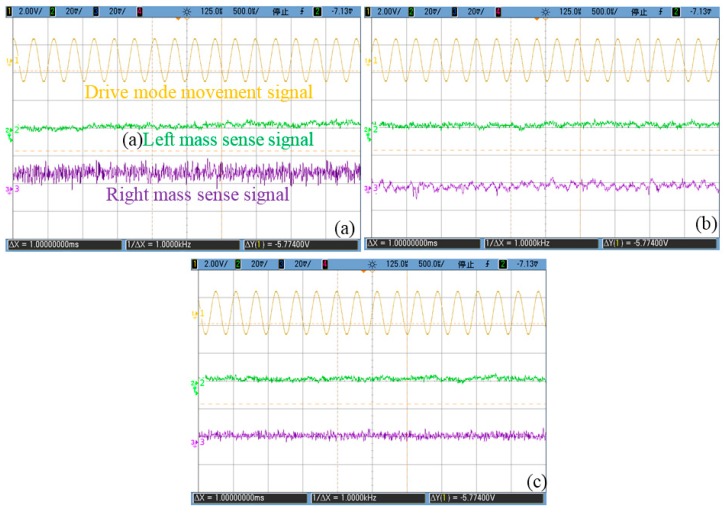
(**a**) CIC system testing results; (**b**) QFC system testing results; (**c**) CSC system testing results.

The experimental equipment and gyro section view is shown in Figure 13. The structure is in a vacuum chip, and the circuits are configured on three PCB boards: PCB I contains the interface and drive closed loop and connects the structure chip; PCB II contains quadrature error demodulator, CIC and CSC systems; PCB III contains the QFC system and sense detection loop. The overall gyro is put in a steel shell, and the experiment is arranged on a turntable inside a temperature oven. The signals are observed with oscilloscope (DSO7104B, Agilent, Santa Clara, CA, USA), and the data is picked up by multimeter (Agilent 34401A) with a sample rate of one point per second. Three corrections are tested separately, and the curves are shown in Figure 14. The three signal curves are drive mode movement signal *V_QED_*cos(*ω_d_t + φ_e_ + φ_n_*), the left mass sense signal *V_LQS_* and right mass sense signal *V_RQS_*. Figure 14a is the CIC system testing result curves, and they show that *V_LQS_* and *V_RQS_* do not contain obvious quadrature error signals, which proves the theory analysis in Section 3.2. QFC system testing results are shown in Figure 14b, and the noise is the main characteristic of *V_LQS_* and *V_RQS_*, which means the quadrature error force is basically corrected. The CSC system testing results are shown in Figure 14c, and the curves indicate that the quadrature error is corrected. Furthermore, it can be concluded that the noise performance of CSC system is better than that of CIC and QFC, especially in *V_RQS_*.

**Figure 15:**
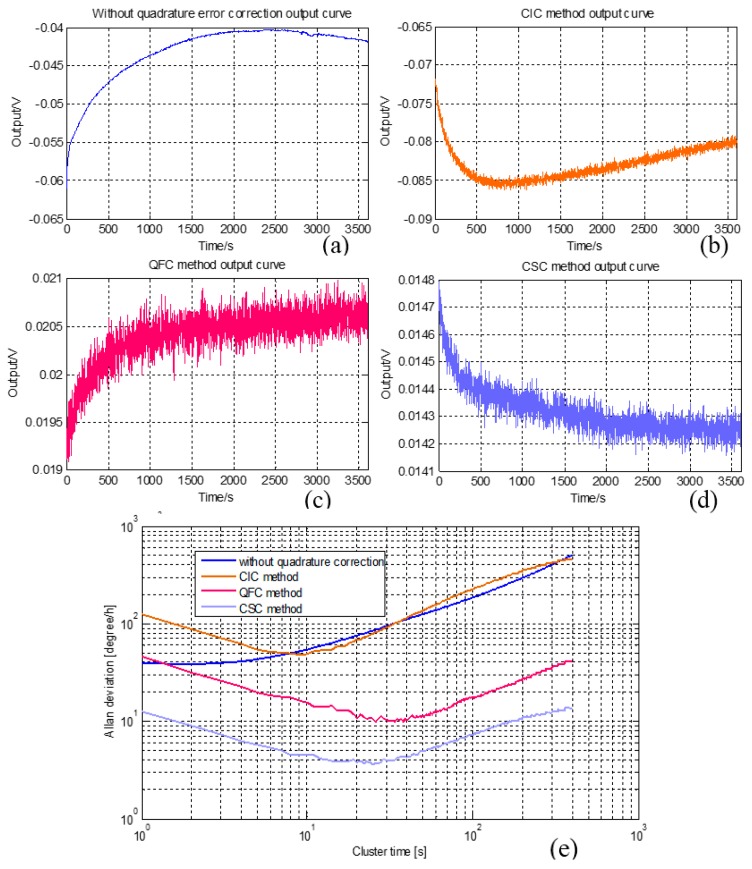
(**a**) Output signal without quadrature error; (**b**) Output signal with CIC system; (**c**) Output signal with QFC system; (**d**) Output signal with CSC system; (**e**) Allan deviation curves.

The output signals of the three methods are also tested, and the results are shown in Figure 15. The output signal without quadrature error correction is shown in Figure 15a, where the curve drift trend is obvious and the bias stability is 38 °/h. Figure 15b shows the output signal after employing the CIC method. Since the quadrature error movement still exists, the drift trend is not optimized and the bias stability is 48 °/h. The QFC method focuses on the quadrature error force, and reduces bias drift trend distinctly (as shown in Figure 15c). Its bias stability improves a lot and achieves 9.9 °/h. The most effective quadrature error correction method is CSC, which can be proven by the drift trend curve. The bias stability of SCS is 3.7 °/h. So, this work considers that the CSC method is the optimal method for quadrature error correction. The general tests of the CSC system are outlined in the next section.

## 5. CSC System General Tests 

**Figure 16:**
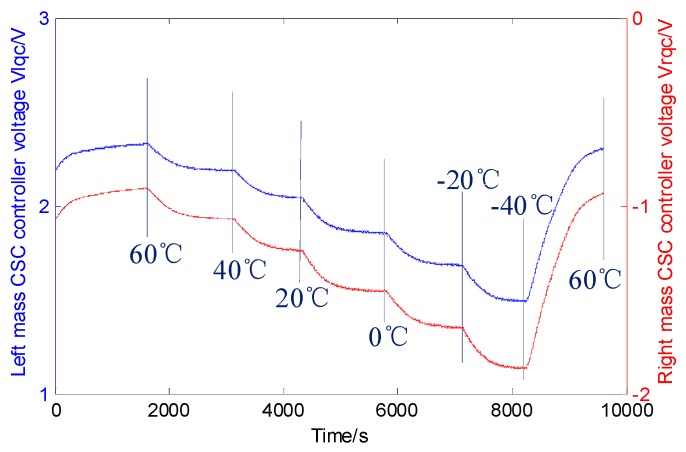
Full temperature range test results.

This section outlines the CSC system tests to inspect the full-temperature range stability and bias repeatability. The temperature test is arranged, the temperature is ranged from −40 °C to 60 °C, and the left and right masses’ CSC controller signals are observed (as shown in Figure 16). The gyro is warmed up to 60 °C, and the temperature is maintained for 20 min. Then, the gyro is cooled in 20 °C increments, until its temperature decreases to −40 °C, and after 20 min, it is warmed up to 60 °C again. During the process, *V_lqc_* and *V_rqc_* change with temperature, which means that the quadrature error coupling stiffness varies, and proves the inference in Section 2. 

**Figure 17:**
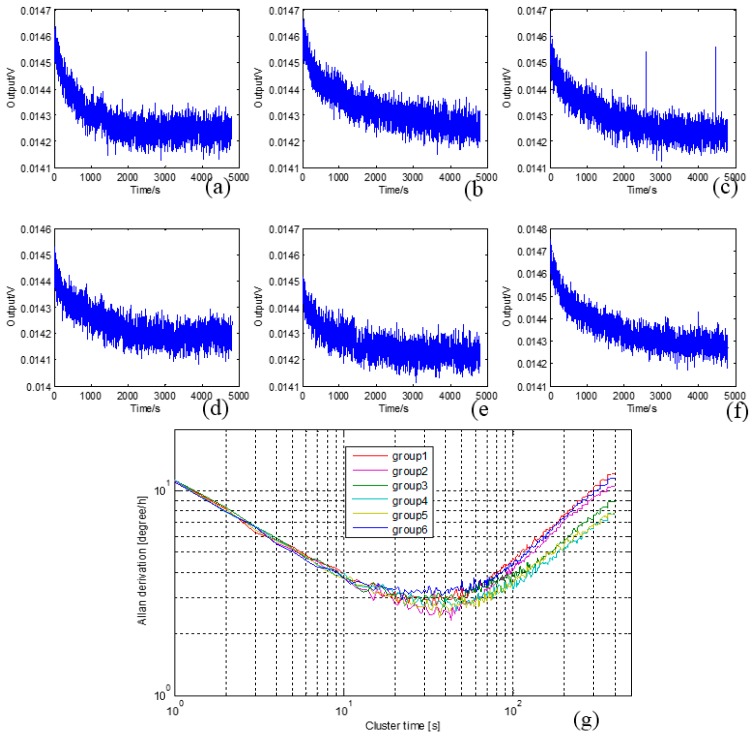
(**a**)–(**f**) Six groups’ CSC system bias repeatability test curves; (**g**) Allan derivation of the six groups’ curves.

Furthermore, the continuous curves indicate that the left and right CSC systems work well, and the values change by almost 50%. The gyro bias repeatability is tested also (each test continues 4800 s), and the six groups’ test results are shown in Figure 17. 

**Figure 18:**
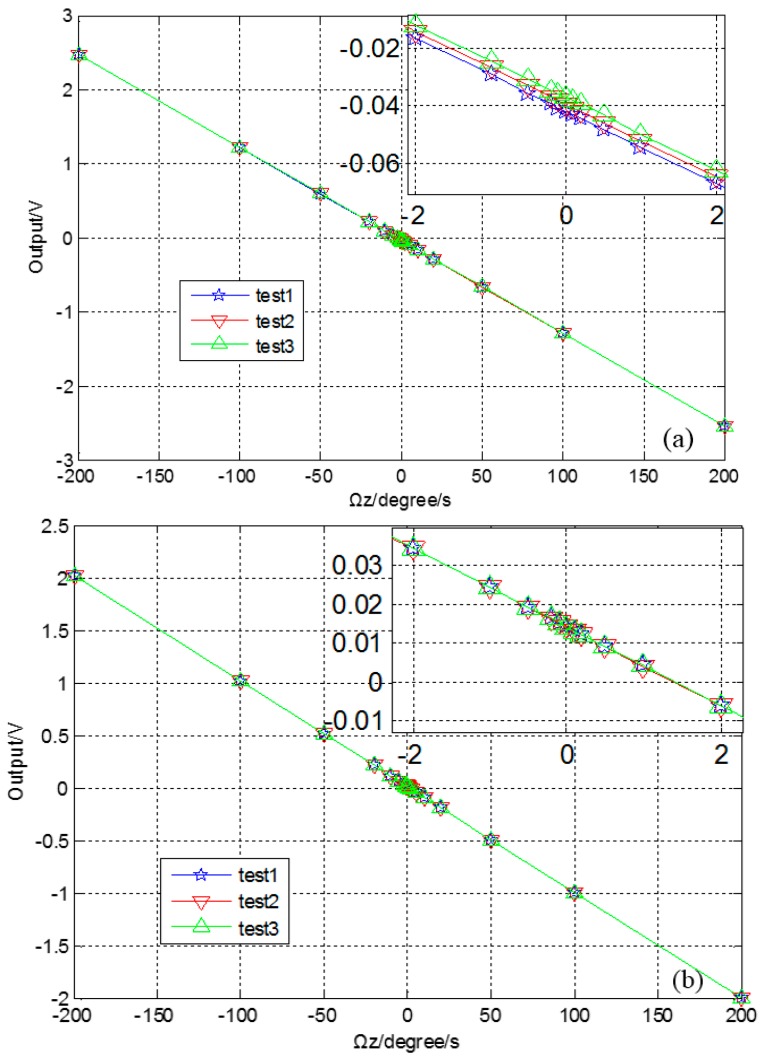
Scale factor test, without quadrature error correction (**a**) and CSC method (**b**).

The results prove that the CSC method has excellent repeatability. The turntable tests (shown in Figure 18) are arranged with input angular rates *Ω_z_* of ±0.1, ±0.2, ±0.5, ±1, ±2, ±5, ±10, ±20, ±50, ±100, ±200 °/s. Three repeat tests shows that the scale factor nonlinearity and repeatability are both improved with the CSC method. The results are summarized in Table 2. 

**Table 2:** Test summary.

**System**	**Parameter**	**Value**	**CSC Test Group**	**Parameter**	**Value**
Without correction	Bias value	4.16 °/s			
	Bias stability	38 °/h			
	ARW	0.66 °/√h	1	Bias value	1.747 °/s
	Scale factor nonlinearity	925 ppm		Bias stability	3.8 °/h
	Scale factor repeatability	818 ppm	2	Bias value	1.752 °/s
CIC method	Bias value	7.92 °/s		Bias stability	3.6 °/h
	Bias stability	48 °/h	3	Bias value	1.748 °/s
	ARW	2.07 °/√h		Bias stability	3.4 °/h
QFC method	Bias value	2.26 °/s	4	Bias value	1.741 °/s
	Bias stability	9.9 °/h		Bias stability	3.1 °/h
	ARW	0.77 °/√h	5	Bias value	1.744 °/s
CSC method	Bias value	1.749 °/s		Bias stability	3.0 °/h
	Bias stability	3.7 °/h	6	Bias value	1.756 °/s
	ARW	0.21 °/√h		Bias stability	4.2 °/h
	Scale factor nonlinearity	660 ppm			
	Scale factor repeatability	403 ppm			

## 6. Conclusions

This paper explores the most optimal quadrature error correction method for the dual-mass gyroscope. Based on quadrature error correction technology, the MEMS gyro’s long term drift can be efficiently suppressed, and the stability, and drift performance of the gyro are improved. Meanwhile, the structures with great coupling stiffness can achieve higher precision and demonstrate improved structure yields.

In this work, the dual-mass structure is introduced, and the coupling stiffness system model is proposed, showing that the quadrature errors are different in the two masses. This theory is proved by experiments, suggesting that quadrature correction should utilize separate correction methods for the two masses. Then, the process that leads to quadrature error is analyzed, and is divided into three stages: coupling stiffness, quadrature error force and quadrature error signal. For these three stages, this work employs the charge injection correction (CIC) method, the quadrature error force correction (QFC) method and the coupling stiffness correction (CSC) method, respectively, to eliminate quadrature error within the different stages. The correction principles of each method are analyzed, and the system stabilities are assessed and simulated. Since CIC and QFC need to be modulated with quadrature error in-phase signals, the phase error and noise introduce more error to the system, and the CSC method avoids this process. Also, the CSC method targets the coupling stiffness source, so it should achieve better correction results.

Finally, experiments are conducted. The curves show that every method can effectively mitigate the quadrature error, and the CSC method achieves better noise characteristics. The output signal before quadrature error correction demonstrates obvious drift, and the bias value, stability and ARW are 4.16 °/s, 38 °/h and 0.66 °/√h. The bias value, stability and ARW of CIC, QFC and CSC are 7.92 °/s, 48 °/h, 2.07 °/√h; 2,26 °/s, 9.9 °/h, 0.77 °/√h and 1.749 °/s, 3.7 °/h, 0.21 °/√h, respectively, which show CSC is the best method for this structure, and proves the theoretical analysis. The CSC system is also tested in the range of −40 ℃ to 60 ℃, and the results show that the left and right systems work well. The bias repeatability tests are done, and six groups’ curves prove the repeatability and reliability of the CSC system.
